# The effect of dance interventions on well-being dimensions in older adults: a systematic review

**DOI:** 10.3389/fspor.2025.1594754

**Published:** 2025-07-25

**Authors:** Ingrid Fonseca, María Rueda, Carlos Cabanzo

**Affiliations:** Faculty of Physical Education, National Pedagogical University (UPN), Bogotá, Colombia

**Keywords:** dance, older adults, systematic review, well-being, quality of life

## Abstract

**Background:**

Dance is increasingly recognized as a strategy that can support healthy aging. It incorporates physical, emotional, cognitive, and social engagement, which makes it particularly relevant for older populations. However, the effects of dance on multidimensional well-being have not yet been thoroughly synthesized.

**Objectives:**

Systematically review empirical studies examining the effects of dance-based interventions on physical, emotional, cognitive, and social dimensions of well-being in older adults. We considered studies that assessed one or more of these dimensions as indicators of well-being.

**Data sources:**

Studies were identified through database searches in Scopus, Web of Science, and SportDiscus conducted between October and November 2024.

**Study eligibility criteria, participants, and interventions:**

Included studies were qualitative or quantitative empirical research published in peer-reviewed journals. Participants were adults aged 60 and older or identified as older adults. Interventions involved dance-based activities. Comparators included no intervention or alternative physical or recreational programs. The outcomes addressed at least one domain of well-being.

**Synthesis methods:**

This review followed the PRISMA 2020 guidelines. Eligibility criteria were defined using the PICOS framework. Study quality was assessed using Law et al.'s (1998) 16-item checklist. Due to methodological heterogeneity, a narrative synthesis was performed.

**Limitations and conclusions:**

Although the results suggest that dance is a promising, low-cost intervention for promoting multidimensional well-being in older adults, several limitations should be noted. Many studies had small sample sizes or did not report effect sizes or randomization. Furthermore, some studies assessed only one or two dimensions of well-being rather than a multidimensional profile. This limits the scope of conclusions that can be drawn about integrated well-being. Future research should prioritize more rigorous designs, standardize multidimensional outcome measures, and assess long-term integrative effects to better inform health promotion policies.

## Introduction

The Pan American Health Organization (1) reported that in 2020, 8% of the global population was aged 65 or older, and it is estimated that by 2050, this figure will exceed 30%, posing a global challenge. Similarly, the World Health Organization (2) population aged 60 and above will double, increasing from 12% to 22%. This demographic growth presents a significant challenge, as many elderly individuals may lack the necessary resources to maintain an adequate quality of life.

To address these challenges, international organizations emphasize the fundamental role of recreational activities and physical activity in promoting the quality of life and well-being of the elderly, as they contribute to strengthening their abilities (3).

### Elderly people and well-being

The concept of an elderly person varies depending on cultural context and should not be limited solely to chronological age but should also consider factors such as functional independence (4). During this stage of life, physical, cognitive, social, and emotional transformations take place, which can influence overall well-being and may lead to a decline in mental faculties (5, 6). In this sense, the physical and cognitive decline characteristic of this stage can lead to estrangement in family relationships and, subsequently, in the social sphere. This demonstrates that different areas of life influence well-being (7).

This stage of life represents a process experienced by all people, regardless of gender (8). In this context, the importance of providing specialized care to improve quality of life and generate well-being is emphasized. At the same time, aging presents opportunities for older adults to participate in new experiences and recreational activities, strengthen social relationships, and foster personal development (9).

In this review, well-being is conceptualized as a multidimensional construct that includes physical, emotional, cognitive, and social domains of health represents a holistic condition that encompasses all aspects of health (10). It encompasses both subjective components, such as individual satisfaction and emotional experiences, and aspects, including functional capacity and social connectedness (11, 12). In this regard, it is essential to foster and maintain interpersonal relationships as well as to actively contribute to the community.

Well-being facilitates positive physical and mental experiences, leading to pleasurable and satisfying moments (13). It encompasses economic stability, physical and mental health, personal relationships, community and social participation, and, more broadly, all aspects that contribute to an adequate quality of life (14). In this sense, it involves feeling satisfied and balanced in all areas of life, enjoying healthy relationships, maintaining a good quality of life, and experiencing harmony and fulfillment (10).

### Dance and well-being in the elderly

The WHO recommends that older adults engage in physical activity (PA) through recreational or leisure activities to enhance their physical, emotional, and cognitive health. This approach promotes happiness, vitality, and greater self-esteem while also strengthening their ability to face challenges and adapt to change (15). Dance practice among the elderly is associated with a reduction in symptoms of anxiety and depression, contributing to a more positive perception of life by allowing them to connect with their emotions and express themselves (16, 17).

Specifically, rhythmic movement interventions have demonstrated benefits including improved bone density, circulation, balance, mobility, flexibility, cognitive function, and mood (18). Additionally, it contributes to cardiovascular health and stress reduction, promoting overall well-being (19). Dance has also demonstrated a positive impact on balance, gait, and motor function (20) while regular participation contributes to fall prevention and reduces the need for hospitalization (21). Dance promotes multidimensional well-being, offering benefits for mood, quality of life, and both mental and psychosocial health (22). Consequently, such interventions contribute to well-being by improving physical performance, preventing falls, boosting self-esteem and mood, promoting independence, and fostering a high quality of life (19).

Beyond its individual effects, dance supports active aging by fostering enjoyment, strengthening the sense of connection, and helping to counteract loneliness (23, 24). This approach combines rhythmic and coordinated body movements, typically accompanied by music, integrating movement and melody to facilitate self-expression. In the therapeutic field, it has also proven to be an effective treatment for depression (25, 26).

Recent studies emphasize the importance of implementing structured programs and leveraging technology to overcome barriers such as physical distancing (27, 28). Likewise, research by Hanks et al. (29) highlights that dance helps develop discipline, establish routines, and improve mood. Similarly, significant progress has been made in the use of rhythmic movements in therapies for adults with dementia, enhancing psychosocial interaction (30).

Dance has benefits for the elderly when approached from a multidimensional, holistic, well-being perspective. However, recent evidence has not been systematically reviewed using PRISMA. This systematic review aims to analyze the influence of dance-based interventions on physical, emotional, cognitive, and social dimensions of well-being, considered as integral components of overall well-being, in older adults. We considered studies that assessed one or more of these dimensions as indicators of well-being. This systematic review addresses the following question using the PICOS criteria: In older adults (Population), does participation in dance-based interventions (Intervention), compared to no intervention or alternative physical or recreational activities (Comparison), improvements in at least one of the following dimensions of well-being: physical, emotional, cognitive, or social. (Outcomes), as reported in empirical studies published in peer-reviewed journals (Study Design)?

## Methodology

### Search strategy

This study followed the guidelines established in the PRISMA (Preferred Reporting Items for Systematic Reviews and Meta-Analyses) statement (31). Data collection was conducted between October and November 2024 using the Web of Science, Scopus, and SportDiscus databases. These databases were prioritized in this review due to their broad coverage of interdisciplinary research, particularly in the fields of health and physical activity, in which dance-based intervention studies are frequently published.

For the study search, keywords were used in combination with Boolean operators (“AND” and “OR”), applying the following search strategy:

(“older” OR “older adults” OR “elderly” OR “elder” OR “elders” OR “older person”) AND (Well-being OR “wellbeing”) AND (dance OR danc OR dancing).

The search strategy was guided by the conceptualization of well-being as a multidimensional construct encompassing physical, emotional, cognitive, and social aspects of health. Although our search strategy focused on the term well-being, we also included studies that reported results in at least one of its dimensions (physical, emotional, cognitive, or social), full-text screening was applied to identify studies that addressed this construct.

The selection of eligible studies was guided by the PICOS criteria (population, intervention, comparison, outcome and study design):

• Population (P): Adults generally aged 60 years and older. Studies with a lower age threshold (minimum age of 50) were also considered eligible if participants were identified as older adults and the outcomes addressed age-related dimensions of well-being.
• Intervention (I): Participation in dance-based activities, including therapeutic, recreational, structured or virtual.
• Comparator (C): No intervention or alternative interventions, such as traditional physical exercise or other recreational programs.
• Outcomes (O): Improvements in at least one of the following dimensions of well-being: physical, emotional, cognitive, or social.
• Study Design (S): Original empirical studies (quantitative or qualitative) published in peer-reviewed scientific journals.

The sample size of each included study was recorded during the data extraction process. However, no minimum or maximum sample size was set as an inclusion criterion, due to the exploration nature and methodological heterogeneity of the selected studies. Where reported, sample sizes were described and considered when interpreting the strength and generalizability of the findings. The retrieved results were exported to RefWorks, where duplicate articles were removed. No restrictions were set regarding publication date.

### Eligibility criteria

Studies had to meet the following criteria: (1) be original research articles published in peer-reviewed journals; (2) present original empirical research (quantitative or qualitative) investigating the relationship between dance-based interventions and well-being in older adults; (3) explicitly address well-being by assessing at least one of its dimensions (physical, emotional, cognitive, or social), or by considering it as a holistic construct. This applies regardless of whether the term “well-being” appears in the title, abstract, or full text and (4) no restrictions were applied regarding publication region, language, gender, or participant age.

Those investigations that (1) did not provide a clearly defined methodological design; or (2) were non-original research articles, including conference proceedings, abstracts, books, editorials, reviews, case studies, and doctoral theses were excluded.

### Study selection

Two independent reviewers evaluated the titles and abstracts of the articles to determine their eligibility, removing duplicate documents during the process. The selected articles were then retrieved for full-text review. Each reviewer analyzed the texts individually, and inclusion was determined by consensus. In cases of disagreement, a third reviewer with methodological expertise intervened to resolve discrepancies.

**Figure 1:**
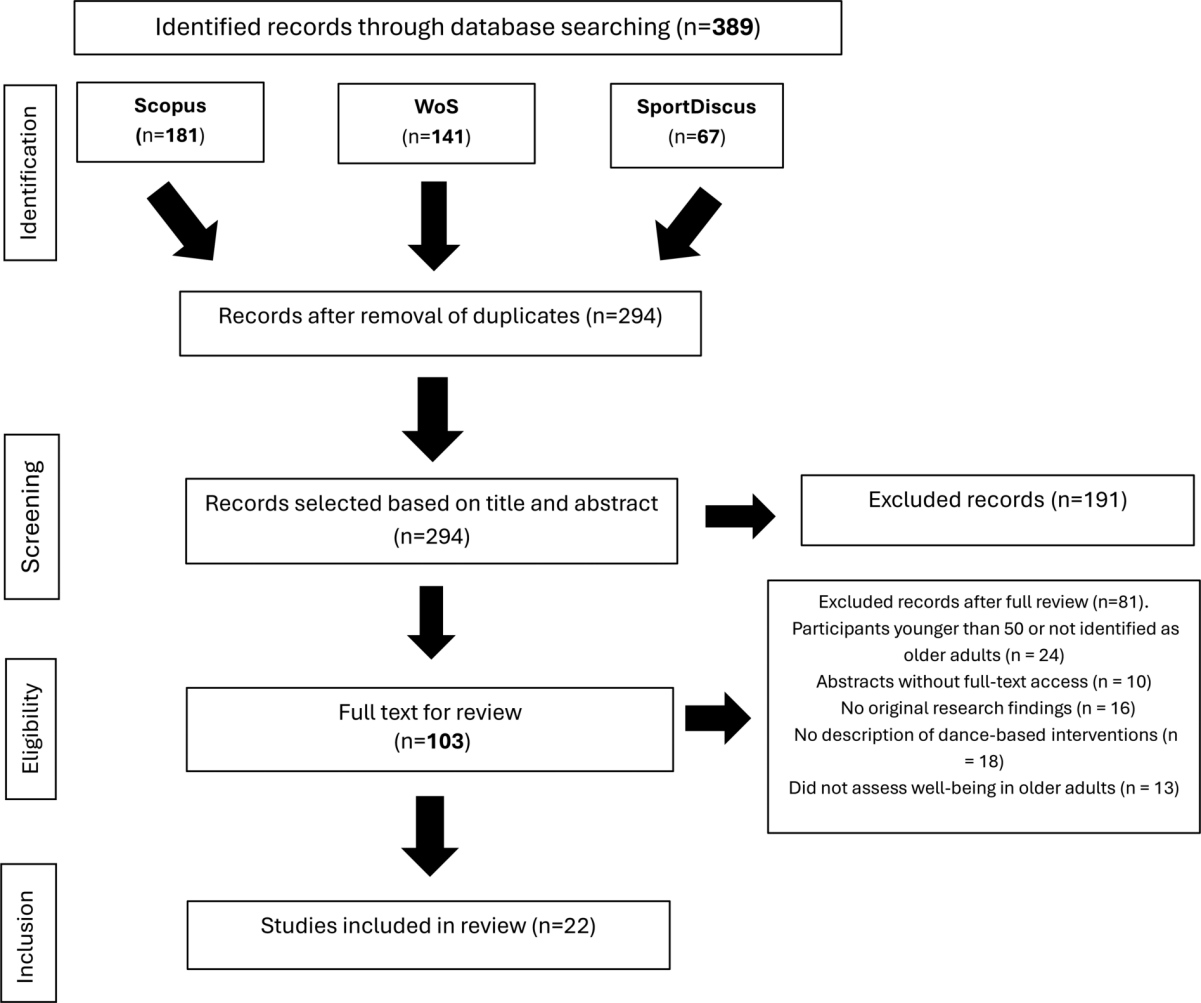
Flow diagram, PRISMA. Source: Prepared by the authors based on PRISMA guidelines.

Figure 1 presents the systematic review process flowchart. The initial literature search yielded a total of 389 records, distributed as follows: Web of Science (141), Scopus (181) y SportDiscus (67).

Subsequently, 95 duplicate articles were excluded, resulting in a total of 294 records. After screening the titles and abstracts, 191 records were excluded because they did not meet the inclusion criteria or were incompatible with the focus of this systematic review. A full assessment was conducted on the remaining 103 articles, and 81 were excluded for the following reasons: 24 included participants younger than 50 or did not identify them as older adults, 10 were conference abstracts without full-text access, 16 did not report original research findings (e.g., reviews, commentaries, or editorials), 18 did not describe dance-based interventions, and 13 did not assess any dimension of well-being in older adults. Finally, 22 studies met all eligibility criteria and were included in the final review.

### Data extraction and quality assessment

The extracted data, recorded in the analysis table, include the following variables: author's name, year of publication, study objective, country, population, number of participants, gender, age, intervention setting, duration, tools used, follow-up, analyzed variables, and main results.

To assess the methodological quality of the studies, the 16-item checklist developed by Law et al. (32) was applied. This tool is designed to evaluate the rigor of the selected studies. Based on this criterion, the following quality categories were established: low quality (score of 50% or lower), good quality (score between 51% and 75%) and excellent quality (score of 75% or higher).

Among the studies analyzed, 16 were classified as excellent quality, 6 as good quality, and 1 received a regular quality rating. The 5 studies classified as low quality were excluded from the review.

## Results

The studies included in this review were conducted across various countries, reflecting a diverse geographical scope. Research was carried out in Australia (33, 34), Brazil (35–37), China (38, 39), South Korea (40, 41), the United States (22, 42–44), Greece (19), India (45), Ireland (46, 47), Italy (48), Portugal (49), and the United Kingdom (50, 51). This broad distribution provides valuable insights into the relationship between dance and well-being in the elderly, incorporating perspectives from diverse cultural and healthcare settings.

In terms of distribution, the United States had the highest number of studies (*n* = 4), followed by Brazil (*n* = 3), which leads among Latin American countries in this field of research. Additionally, Australia, China, South Korea, Greece, and the United Kingdom each contributed two studies, while India, Italy, Portugal, and the Czech Republic had one study each. This distribution highlights the prominence of research on dance and well-being in certain regions while also indicating the need for further studies in underrepresented areas.

### Sample size

The sample sizes in the reviewed studies varied significantly:

Small samples (≤20 participants): 8 participants: Philip et al. (51), Han & Sa (41). Between 10 and 17 participants: Aliberti & Raiola (48) (14), Silva et al. (37) (17), Thumuluri et al. (44) (10).
Medium-sized samples (21–44 participants): Holmerová et al. (52), Aguiñaga & Márquez (43), De Araujo & Da Rocha (36), Im et al. (40), Zhao et al. (39), Chipperfield & Stephenson (50), Harrison et al. (22).
Large samples (52–100 participants): Lima & Vieira (35), Holmerová et al. (52), Cruz-Ferreira et al. (49), O'Toole et al. (46), Lukach (42), Wang et al. (38), Clifford et al. (47), Pandya (45).
Very large samples (127–661 participants): Poulos et al. (33), Douka et al. (19), Waugh et al. (34).

### Population

While most studies focused on the elderly in general, several investigations examined specific populations with conditions, including Parkinson's disease (mild to moderate stages) (45); Cognitive impairment (38, 39, 44); Mobility difficulties (21, 48); Respiratory diseases (51); Post menopause (40); Leisure and cultural activities (36); Women-only groups (34, 40, 41, 49).

Among the 22 studies analyzed, 18 included mixed-gender populations, whereas 4 focused exclusively on women. The most studied population was individuals with physical, functional, or fitness-related difficulties (*n* = 8), including two studies (*n* = 2) on fall prevention. Additionally, four studies (*n* = 4) explored psychological or psychosocial aspects, while nine studies (*n* = 9) addressed mental health. Regarding participant age, the recorded range spanned from 55 to 97 years, establishing this interval as the reference population across the analyzed studies.

Although the inclusion criteria aimed to focus on adults aged 60 years and older, two studies with slightly younger participants (minimum ages of 50 and 55, respectively) were retained. These studies were included because they explicitly identified their participants as older adults or seniors, and the average age of participants fell within the typical older adult range. Additionally, both studies addressed aging-related outcomes relevant to the review, such as physical decline, emotional well-being, and social participation.

## Results by intervention and well-being dimension

Of the 22 studies reviewed, the majority (*n* = 21) assessed two or more dimensions of well-being. Seven studies addressed three or more dimensions: physical, emotional, cognitive, and/or social (22, 33, 35, 36, 38, 47, 51). Fourteen studies assessed two dimensions of well-being; these included Pandya (45); Thumuluri et al. (44); Chipperfield and Stephenson (50); Zhao et al. (39); Aliberti and Raiola (48); Aguiñaga and Márquez (43); Douka et al. (19); Cruz-Ferreira et al. (49); O'Toole et al. (46); Holmerová et al. (52); Han & Sa (41); Silva et al. (37); Waugh et al. (34); and Philip et al. (51). Only one study focused exclusively on the physical dimension (40); however, it was included because it demonstrates improvements in strength, balance, and flexibility—fundamental components of functional well-being in older adults.

Interventions lasting 12 weeks or more, with a higher frequency of two to three sessions per week such as those implemented by De Araujo & Da Rocha (36), Douka et al. (19), Lukach et al. (42), Pandya (45) reported significant improvements in physical function, emotional well-being, mental health and cognitive level. In contrast, programs that were shorter or had a lower frequency [e.g., (46)] primarily reported benefits within emotional and social domains.

The type of dance intervention also influenced the results. Virtual ballet (22) and improvisational dance (44) improved mobility, balance, and cognitive function. Traditional (19, 35), Latin (43), and circle (37) dance interventions were more effective in improving social connectedness, reducing loneliness, and promoting subjective well-being.

Regarding study design, randomized controlled trials and quantitative studies employing pre and post intervention evaluations tended to report more robust and measurable effects. Examples include studies by Douka et al. (19), Clifford et al. (47), Wang et al. (38), Harrison et al. (22), and Im et al. (40). In contrast, qualitative studies [e.g., (36, 37, 51)] provided interpretive insights, though they lacked estimates of effect size. While not all studies reported effect size, those that did, such as those by Cruz-Ferreira et al. (49) and Douka et al. (19), demonstrated clinically significant improvements in strength, balance, and life satisfaction.

All the included studies addressed well-being, either by assessing at least one of its dimensions (physical, emotional, cognitive or social), or by considering it as a multidimensional construct. This was confirmed through a review of the full texts (Table 1).

**Table 1:** Articles reviewed.

No	Author & year	Study's objective	Location	Population & Sample size	Gender	Age	Intervention	Duration	Instruments	Follow up	Measured variables	Well-being dimension	Key findings
1	Pandya (45)	Assess the impact of Dance Movement Therapy (DMT) and yoga (DMT + Y) on balance, geriatric anxiety, and well-being in elderly individuals with Parkinson's disease.	Mumbai, India	74 older adults with mild to moderate Parkinson's disease	38 men (51%), 36 women (49%)	60–79 years	Dance Movement Therapy (DMT) and a combined approach of DMT with yoga (DMT + Y)	6-month intervention with sessions of two different styles	Balance Confidence Scale Geriatric Anxiety Scale (GAS-10) Warwick-Edinburgh Mental Well-being Scale (WEMWBS)	Post-intervention evaluations	Balance, geriatric anxiety, and well-being	Physical, emotional	Significant improvements in balance confidence and a reduction in anxiety, particularly among older women who regularly participated in the DMT + Y program.
2	Clifford et al. (47)	Assess the feasibility of a music and dance program for elders living in the community, as well as its impact on health and well-being.	Ireland	100 elders living in the community	87 women (87%), 13 men (13%)	Mean age: 73.73 years (SD = 5.61) for the intervention group, 74.18 years (SD = 4.95) for the control group	Music and Movement for Health (MMH) Program	6 weeks with 2 weekly sessions, each lasting 1.5 h	Incidental and Planned Exercise Questionnaire (IPEQ) Timed Up and Go Test (TUG) 30-Second Chair Stand Test (30CST) Short Physical Performance Battery (SPPB)	Post-intervention evaluation	Physical activity, physical performance, functional mobility, balance, and psychosocial well-being	Emotional, physical, social	The study demonstrated that intervention had a positive impact on elder health. All predetermined feasibility criteria were met, and the program was suggested to have the potential for cost-effectiveness.
3	Harrison et al. (22)	Evaluate the effectiveness of virtual ballet classes compared to virtual Well-being classes, focusing on improving mobility, balance, and quality of life in elderly women. The study aimed to quantify the benefits of virtual ballet and address underserved populations in this context.	St. Louis Missouri, United States	44 elders from the community	44 women (100%)	Mean age: 67.81 years for the Ballet group, 69.96 years for the Well-being group	Virtual modified ballet classes for elders compared to control Well-being-based virtual classes	12 weeks, with two sessions per week, each lasting 45 min	Portable inertial sensors for gait and balance assessments Self-report questionnaires Quality of life and mood evaluations Two-minute Walk test Timed Up and Go (TUG)	Pre- and post-intervention evaluations	Gait assessment, walking speed, balance, quality of life, fall rate	Emotional, physical, social	Virtual ballet classes significantly improved mobility, balance, and overall quality of life in elderly women compared to Well-being classes. While both interventions enhanced gait and balance, the ballet group demonstrated superior benefits, particularly in backward gait variability and a significant reduction in fall rates. These findings highlight ballet's effectiveness as an intervention for improving functional mobility in this population.
4	Hans & Sa (41)	Investigate the effect of elderly women's participation in dance activities on their quality of life, health, and happiness.	South Korea	8 elderly women participating in a dance program	8 women (100%)	59–79 years	Participation in a dance program	3 months	In-depth interviews Participant observations	Post-intervention evaluations	Health, happiness, life satisfaction, motivations for participating in activities	Social, emotional	The results confirmed the significance of health and happiness feelings induced by dance among elderly women. The study suggests that engaging in dance activities can enhance their quality of life and contribute to successful aging.
5	Thumuluri et al. (44)	Evaluate the effects of improvisational movement therapy on quality of life, balance, mood, and brain connectivity in individuals with early-stage dementia or mild cognitive impairment.	Winston-Salem, North Carolina, United States	10 individuals with mild cognitive impairment or early-stage dementia	Men and women (no specific breakdown)	60–90 years	Improvisational dance classes using the IMPROVment® method	8 weeks, 2 sessions per week	Balance evaluations (Fullerton Advanced Balance Scale) Quality of life (QoL-AD) Mood assessment (Geriatric Depression Scale)	Conducted at the end of the 8-week intervention	Quality of life, balance, mood, and brain connectivity	Cognitive, physical, emotional	Significant improvements were observed in quality of life and balance, while mood showed no notable change. Brain connectivity also demonstrated increased global efficiency.
6	Chipperfield & Stephenson (50)	Investigate the effect of social ballroom dancing on well-being, balance, and fall prevention in elders living in the community.	United Kingdom	26 individuals participating in ballroom dance classes at least once per week	Women: 54% (*n* = 14) Men: 46% (*n* = 12)	58–83 years, with a mean age of 66.7 years	Ballroom dance sessions at least once per week for a minimum of one hour	12 months	Routine health assessment (CORE-GP) International Falls Efficacy Scale (FES-I) Timed Up and Go Test (TUGT) Biodex Global Stability Index	Physical assessments conducted at baseline, 3 months, 6 months, 9 months, and 12 months	Age, weekly dance participation, BMI, self-reported falls in the previous 12 months	Emotional, physical, social	Participants engaged in ballroom dancing maintained an active lifestyle, had lower BMI levels compared to the general population in England, and demonstrated strong adherence to the activity. Significant improvements were observed in balance, and there was a reduced risk of falls.
7	Silva et al. (37)	Understand circle dance as an integrative and complementary practice for promoting health in the daily lives of elders.	Brazil	17 elders	Women: 99% (*n* = 16) Men: 1% (*n* = 1)	64–82 years	Weekly circle dance sessions	3 months	Schatzman and Strauss’ suggested model Consolidated Criteria for Reporting Qualitative Research (COREQ)	Conducted before, during, and after each intervention	Interactions, meanings, collective experiences	Social, emotional	Circle dance provided a sense of belonging, pleasure, and well-being among elders, contributing to the promotion of their overall health.
8	Zhao et al. (39)	Explore the acceptability and feasibility of public square dancing among elders in the community with mild cognitive impairment (MCI) and depressive symptoms.	Tiancun Community, Haidian District, Beijing, China	35 elders aged 60–85 with mild cognitive impairment and depressive symptoms	Women: 100%	60–85 years	Public square-dancing sessions held three times per week	3 months (sessions of 60 min, three times per week)	Subjective Exercise Experience Scale (SEES) Heart Rate Monitoring	Conducted three months after the intervention, during which participants continued dancing, and psychological well-being.	Psychological well-being, psychological distress, fatigue, heart rate	Emotional, physical, social	Participants reported increased confidence and a sense of achievement. They found public square dancing easy to learn, relevant to their daily lives, and beneficial in fostering a sense of belonging. The intervention enhanced both physical and mental health.
9	Waugh et al. (34)	Evaluate the effectiveness of dance trials among elderly participants.	New South Wales, Australia	530 elders from the community	Women: 450 (85%) Men: 80 (15%)	72–96 years (DAnCE and Falls Trial) 60–86 years (Dancing Minds Study)	Dance trials assessing lifestyle benefits, psychological mediation, physical and cognitive health	10 weeks	Geriatric Depression Scale (GDS) Additional methods to assess well-being	Monitoring conducted throughout the trial period to assess physical and cognitive health interventions.	Self-efficacy, mental health, cognitive state	Cognitive, emotional	The study found that prior dance experience improves self-efficacy in dance among elderly individuals. While correlations were observed between self-assessed dance skills and overall health measures, these were weaker than anticipated. Variations in mental health and physical activity were identified as potential influences on self-efficacy.
10	Aliberti & Raiola (48)	Assess the effects of online dance on mental health, specifically in reducing depression among elderly individuals with mobility difficulties after the COVID-19 pandemic.	Italy	14 elderly individuals with mobility difficulties, specifically Italian senior.	Women: 14 (100%)	65 years	Online dance course (LD) for three months	3 months, with dance sessions three times per week	15-item Geriatric Depression Scale (GDS) Paired-sample T-tests	Evaluation conducted before and after the three-month course.	Depression, life satisfaction, interest in activities, mood, happiness, life perception.	Cognitive, emotional	Participants showed a significant reduction in depression levels. They also reported greater life satisfaction, increased interest in activities, improved mood, and higher happiness levels following the intervention.
11	Philip et al. (51)	Explore the perceived experiences and health impacts of participating in a community dance group for individuals with chronic respiratory diseases (CRD).	Community health center in London, United Kingdom	8 Individuals with chronic respiratory diseases, including COPD, and asthma,	Women: 6 (75%) Men: 2 (25%)	57–87 years (average age: 75 years)	Weekly 75-min community dance sessions tailored for individuals with respiratory difficulties, led by a trained instructor.	2 years	Semi-structured interviews Thematic analysis	Not explicitly mentioned	Health impacts (physical, psychological, social), including physical condition, psychological well-being, and social cohesion.	Emotional, physical, social	Participants reported holistic benefits, such as improved physical fitness, enhanced psychological well-being, reduced need for medical care, and stronger social connections. Practice became an integral part of their lives, providing enjoyment and fostering social engagement.
12	Wang et al. (38)	Explore the effects of modified Chinese square dancing on global cognition, depressive symptoms, balance, and quality of life in older adults with mild cognitive impairment.	Large nursing home in Changchun, China	66 elders with mild cognitive impairment	Women: 47 (71.2%) Men: 19 (28.79%)	Average age: 81.08 years	Modified Chinese square dancing adapted to prevent falls, performed three times per week for 12 weeks.	12 weeks	Cognitive evaluations using MMSE and MoCA Depressive symptom assessment via Geriatric Depression Scale (GDS-15) Balance assessment using the Berg Balance Scale (BBS)	Results were assessed at the beginning of the study and at weeks 6 and 12.	Global cognition, depressive symptoms, balance, and quality of life.	Physical, emotional, cognitive	Chinese square dancing had positive effects on cognition, depressive symptoms, balance, and overall quality of life. Significant improvements were observed in depressive symptoms and mental well-being, particularly by week 12.
13	De Araujo & Da Rocha (36)	Understand the meanings that elders attribute to two group leisure activities: dance classes and musical gatherings.	Rio de Janeiro, Brazil	32 elders (16 in the dance group, 16 in the musical gatherings group).	Women: 9 (56.25%) Men: 7 (43.75%)	60–83 years	Participation in dance classes and musical gatherings.	6 months with weekly meetings	Participant observation In-depth interviews Ethnographic analysis	Conducted during and after the intervention.	Well-being, hedonism, social connection, identity formation, learning.	Emotional, physical, social	Group leisure activities such as dance and musical gatherings enhance well-being, foster social connections, and strengthen identity in elders. Additionally, dance encourages rediscovery of the body, while music evokes nostalgia. These activities help redefine aging as an active and meaningful stage of life.
14	Douka et al. (19)	Investigate the impact of a traditional Greek dance program on functional capacity and well-being in elders aged 60 and above.	Thessaloniki, Greece	130 elders	Women: 107 (82.31%) Men: 23 (17.69%)	Average age of 67 years	Participation in a traditional Greek dance program twice a week for 32 weeks.	32 weeks	Fullerton Senior Fitness Test Single Leg Balance Test Handgrip Strength Test	Evaluations conducted at the beginning and end of the intervention.	Functional capacity, balance, and strength.	Physical, emotional	The study revealed a significantly positive impact on elders’ physical capacity and well-being. After 32 weeks of participation, they showed marked improvements in strength, balance, and flexibility. Tests indicated progress in areas such as grip strength and balance. Overall, the dance program promoted active aging and enhanced quality of life.
15	Im et al. (40)	Evaluate the effectiveness of dance as a therapeutic intervention to improve physical and psychological condition in elders.	Community gymnasium, South Korea	34 Korean women (postmenopausal for at least five years)	Women: 34 (100%)	Control group: 69.36 years Exercise group: 71.57 years	Combined exercise program incorporating yoga and traditional Korean dance.	12 weeks, three sessions per week.	Flexibility, balance, and muscle strength evaluations Bioelectrical impedance analysis (InBody 520) to measure body composition.	Evaluations conducted at the beginning and after 12 weeks.	Changes in body composition, muscle strength, flexibility, and balance.	Physical	Significant improvements were observed in balance, flexibility, and muscle strength after the intervention. However, there were no significant differences in body weight or muscle mass between the beginning and end of the study.
16	Poulos et al. (33)	Evaluate the impact of the “Arts on Prescription” program on improving well-being and health among elderly people with a wide range of health and Well-being needs.	Community of Sydney, New South Wales, Australia.	127 elders living in the community with various health and Well-being needs.	Women: 94 (74%) Men: 33 (26%)	65–96 years (mean age: 78.1 years)	Courses in visual arts, photography, dance, theater, singing, and music taught by professional artists.	8–10 weeks, with weekly classes.	Pre- and post-course questionnaires Focus groups and individual interviews. Warwick-Edinburgh Mental Well-being Scale (WEMWBS)	Evaluations conducted before and after the course.	Mental well-being, frailty, creativity, frequency of creative activities, social connection.	Physical, emotional, cognitive, cognitive	The “Arts on Prescription” program significantly improved mental well-being, creativity levels, and the frequency of creative activities. Additionally, it increased social connections and overall life satisfaction.
17	Aguiñaga & Márquez (43)	Investigate the feasibility and safety of a Latin dance program for Spanish-speaking Latino elders with mild cognitive impairment (MCI).	Adult Well-being center in Chicago, United States.	21 Latino elders with mild cognitive impairment	Women: 16 (76.13%) Men: 5 (23.87%)	Mean age: 75.4 years	“BAILAMOS” Latin dance program, twice per week for 16 weeks.	16 weeks	Evaluation of reach, retention, attendance Dance records Focus groups	Post-intervention evaluations in week 17 and follow-up with the control group.	Quality of life, adherence to the program, physical and cognitive aspects.	Cognitive, emotional, social	The program had a positive impact on mental well-being, fostering artistic activities that create a sense of purpose, enabling personal growth and achievement.
18	Lukach et al. (42)	Describe the protocol of a multicenter study examining the effects of an African dance intervention on cognition, mood, quality of life, and physical fitness in a sample of African American elders.	Community centers in Pittsburgh and Philadelphia, United States.	80 African American elders.	Women: 40 (50%) Men: 40 (50%)	65–75 years	African dance program.	24 weeks	Self-reported demographic data Current and past smoking history Family history of age-related cognitive issues VO2 Submaximal test	Evaluations conducted at the beginning of the intervention and two weeks after completion.	Cognitive function, mood, quality of life, and physical fitness.	Physical, emotional, cognitive	The REACT program assessed the feasibility of being used as a moderate-intensity physical activity intervention for African American elders in a randomized controlled trial. The results indicated that it has the potential to transform community-based interventions and serve as a platform for evaluating similar programs in other populations.
19	Cruz-Ferreira et al. (49)	Evaluate the effects of creative dance on physical fitness and life satisfaction in elder women.	Health center in Évora, Portugal.	57 elder women (32 in the experimental group, 25 in the control group).	Women: 57 (100%)	65–80 years	Participation in a creative dance program for 24 weeks, with three 50-min sessions per week.	12–24 weeks	Senior Fitness Test to evaluate physical fitness Life Satisfaction Scale	Evaluations conducted at baseline, at 12 weeks, and at 24 weeks.	Strength, flexibility, aerobic endurance, agility, balance, and life satisfaction.	Physical, emotional	Women in the intervention group showed significant improvements in all physical fitness dimensions and life satisfaction compared to the control group.
20	O'Toole et al. (46)	Examine the impact of a six-week dance program on activity participation frequency, fall prevention efficacy, and quality of life in elders over 50 living in the community.	Ireland	62 elders living independently in the community.	Women: 57 (91.1%) Men: 5 (8.9%)	Over 50 years	Dance sessions incorporating various musical styles, including jazz and classical pieces.	6 weeks	Frenchay Activities Index Falls Efficacy Scale EQ-5D-3l to assess quality of life	During the final week, a study was conducted on active participation and lifestyle changes.	Activity participation frequency, fall prevention efficacy, quality of life.	Physical, emotional	Significant increase in activity participation, no significant changes in fall prevention efficacy or quality of life. However, participants reported a positive perception of the experience.
21	Holmerová et al. (52)	Evaluate the impact of a seated dance intervention among elders with depressive symptoms living in residential care facilities.	Czech Republic – Residential care facilities in an urban area of Prague.	27 elders classified as sedentary with low functionality, living in residential care.	Women: 25 (92.52%) Men: 2 (7.41%)	Average age: 81 years (= 9.7).	EXDASE seated dance program, with 75-min sessions once a week.	3 months.	Lower body function tests Barthel Index for Activities of Daily Living (ADL) Mini-Mental State Examination (MMSE).	Evaluations conducted before and after the intervention.	Physical functioning, mobility, depressive symptoms.	Emotional, physical	The study results indicate that sedentary elders with low functionality in advanced age can improve lower body function even through relatively simple dance-based exercises.
22	LLima & Vieira (35)	Explore the therapeutic benefits of ballroom dancing for elders and its impact on physical, mental, and social well-being.	Ballroom dance classes in Viçosa, Brazil.	60 elders of Brazilian nationality.	Women: 54 (90%) Men: 6 (10%)	Over 60 years old.	Ballroom dance classes twice per week.	1 year.	-Participant observation -Qualitative questionnaires on the benefits of dance.	No formal follow-up specified, but measurements were taken at the end of the year.	Physical well-being, mental health, social connections, quality of life.	Physical, emotional, social	Significant improvements in physical and mental health, increased flexibility, coordination, and emotional well-being, as well as enhanced social interaction.

The 22 studies included in this systematic review exhibit significant diversity in terms of the types of intervention, the characteristics of the participants, and the dimensions of well-being that are assessed. Despite this diversity, the analysis identifies similarities and differences that are relevant, as well as methodological strengths and limitations.

Similarities among the studies include the consistent reporting of positive effects on at least one dimension of well-being, particularly in the physical, emotional, or social domains. Regardless of the specific dance style or the duration of the intervention, commonly reported benefits include enhanced self-esteem, increased vitality, and improved social interaction. Moreover, dance is presented as an accessible and culturally adaptable strategy that encourages active participation and adherence among older adults.

Differences were observed in the types of dances employed, which ranged from traditional, African, and Latin styles to improvised, ballet, and virtual formats. Intervention durations varied from eight weeks to over one year, with session frequencies ranging from one to three sessions per week. Participants' health statuses also differed across studies: while some studies focused on healthy older adults, others included individuals with Parkinson's disease, cognitive impairment, or mobility limitations. These methodological and contextual differences influenced the reported outcomes and posed challenges for direct comparisons between studies.

From a methodological standpoint, and in accordance with the 2020 PRISMA guidelines, several limitations were identified. Most studies involved small sample sizes (fewer than 44 participants), which limits the generalizability of the findings. Additionally, many studies lacked control groups or randomized designs, thereby constraining the ability to draw causal inferences. A further limitation was the lack of standardized assessment tools, which impeded cross-study comparability. Furthermore, a limitation of this review is that the search strategy was based on the term “well-being”, without including specific descriptors for each dimension (social, cognitive, emotional, and physical), although the full text addresses well-being. Future reviews could broaden the scope of terms used to encompass a wider spectrum of multidimensional well-being.

## Discussion

This systematic review analyzed 22 studies that investigated the effects of dance-based interventions on the multidimensional well-being of older adults. The findings suggest that dance positively influences several interconnected domains, including physical, cognitive, emotional, and social health, and that these improvements frequently occur simultaneously. Our analysis suggests that enhancements in one domain often coincide with changes in others through a holistic process that promotes healthy aging.

We review the types and duration of dance interventions implemented in the studies, the recorded benefits in the physical, cognitive, emotional, and social domains, and the methodological approaches used. Additionally, we discuss recurring limitations, such as small sample sizes and a lack of standardization.

## Contributions of dance to the multidimensional well-being of the elderly

Studies demonstrate that dance-based interventions contribute to the well-being of older adults because the physical, cognitive, emotional, and social benefits are interrelated from a multidimensional perspective considering simultaneous contributions. It should be noted that improvements in one area can reinforce changes in others. However, many studies did not assess well-being as a comprehensive or holistic concept.

Multiple studies conclude that dance has a significant impact on the physical health of the elderly, enhancing strength, flexibility, balance, endurance, and mobility. These benefits contribute to a reduced risk of falls and promote active and independent aging (19, 22, 40, 42, 44, 46, 47, 52). Additionally, Cruz-Ferreira et al. (49) and Lima & Vieira (35) emphasize that dance not only strengthens muscles and improves cardiovascular endurance but also supports cardiorespiratory health. Likewise, Aliberti & Raiola (48) highlights its role in preventing metabolic diseases.

From a preventive perspective, Han & Sa (41) and Wang et al. (38) emphasize that dance practice enhances flexibility, balance, and muscular strength, which helps prevent chronic diseases and promotes overall better health. Additionally, Im et al. (40) highlights that both Korean dance and yoga contribute to hormonal stability, strengthen muscles, and significantly reduce the risk of falls.

Beyond its physical benefits, dance also has a positive impact on mental health. Lukach et al. (42) observed that the rhythmic execution of African dance styles enhances neurocognitive health and reduces the risk of dementia in elderly African Americans. This body-mind connection is also highlighted by Pandya (45), who emphasizes that the combination of dance and yoga improves balance and reduces anxiety in individuals with Parkinson's disease. Similarly, Thumuluri et al. (44) points out that improvisational dance fosters brain connectivity in elderly individuals with early-stage dementia.

The emotional impact of dance is another well-documented aspect. Chipperfield & Stephenson (50) highlight that participating in social activities such as dance helps maintain healthy self-esteem levels and reduces depression. Similarly, O'Toole (46) emphasizes that this practice enhances confidence and mood, providing a sense of vitality and energy. Along the same lines, De Araujo & Da Rocha (36) assert that dance induces pleasure and joy, reinforcing personal identity and fostering an active and fulfilling lifestyle. Additionally, Waugh (34) and Silva (37) concur that dancing reduces stress, improves self-esteem, and strengthens the sense of belonging.

Philip et al. (51) and Zhao et al. (39) emphasize dance's ability to promote overall well-being by enhancing both physical and emotional health while also facilitating social interaction. For elderly individuals with mild cognitive impairment, Aguiñaga & Márquez (43) highlight that Latin dance is particularly effective in generating happiness and energy. Similarly, Cruz-Ferreira et al. (49) stress that dance encourages socialization and contributes to mental well-being.

Dance serves as a key tool for fostering social interaction and reducing feelings of loneliness in old age. Clifford et al. (47) and Harrison et al. (22) highlight its positive impact on psychosocial well-being, as it encourages interpersonal connections and reduces social isolation. In this regard, Aliberti & Raiola (48) emphasize the role of online dance during the COVID-19 lockdown, as it allowed the elderly to remain active and socially engaged in a safe environment.

Beyond immediate social interaction, dance reinforces a sense of purpose and empowerment. O'Toole et al. (46) and Poulos (33) highlight that artistic activities centered on dance help strengthen social relationships and boost self-confidence. Recreational dance practice generates positive emotions by fulfilling needs for autonomy, competence, and belonging. Meanwhile, Wang et al. (38) underscores its role in enhancing cognition and reducing depressive symptoms.

The creation of positive social environments is another notable outcome of dance practice. Aguiñaga & Márquez (43), Poulos (33), Silva (37), and Thumuluri (44) agree that these activities foster an enjoyable social atmosphere, strengthening connections among participants and establishing emotional support networks. Collectively, these studies demonstrate that dance not only enhances physical and mental health but also reinforces a sense of community, contributing to a healthier and more fulfilling aging process.

Related to emotional health, multiple studies support the idea that dance has positive effects. Chipperfield & Stephenson (50) and Harrison et al. (22) indicate that this activity reduces anxiety and boosts self-esteem, while Lima & Vieira (35) and Cruz-Ferreira et al. (49) emphasize its ability to enhance mood and alleviate stress.

Besides, from a therapeutic standpoint, Roca-Amat & García-Alandete (17) highlight that dance plays a fundamental role in emotional expression, offering a space for personal development and psychological well-being enhancement. Moreover, its impact transcends cultures and diverse populations, establishing itself as a universal tool for emotional well-being in old age.

In terms of cognitive perspective, dance has been linked to improvements in attention and executive function. Clifford et al. (47) indicates that dance enhances mental agility and supports the preservation of essential cognitive skills. Meanwhile, Im et al. (40) and Zhao et al. (39) emphasize that memorizing dance steps improves concentration and strengthens memory.

Additionally, Douka et al. (19) and Thumuluri et al. (44) reinforce these findings, demonstrating that dance positively impacts overall cognitive function, particularly in maintaining memory and focus. Finally, Aliberti & Raiola (48) highlights that this practice activates key brain regions involved in learning and information retention, establishing itself as an effective strategy for enhancing cognitive functions in the elderly.

## Influence of intervention characteristics

The following section provides an extensive overview of the types and durations of dance interventions, as well as the research designs and methodological strategies employed by the included studies. The goal of this synthesis is to explore how these elements interact and contribute to the observed effects on the multidimensional well-being of older adults.

### Intervention

The interventions analyzed in the reviewed studies exhibit significant diversity in terms of duration, type of dance, and session frequency. Some studies implemented short-term programs, while others were designed to evaluate the impact over the course of weeks or months.

For instance, in the study by Chipperfield & Stephenson (50), participants attended ballroom dance sessions once per week for one hour. Similarly, O'Toole et al. (46) evaluated a six-week program led by instructors, which included ten-minute warm-up and cooldown sessions. Along the same lines, Clifford et al. (47) analyzed the impact of the Music and Movement for Health (MMH) program, which lasted two months with weekly 90-min sessions, conducted between February-March for participant recruitment and August-September for the intervention.

Other studies implemented medium-duration interventions, structured over several weeks. In the case of Harrison et al. (22), the study analyzed modified virtual ballet classes for the elderly, with 45-min sessions twice a week for 12 weeks, compared to control classes focused on general well-being. Similarly, Han & Sa (41) and Wang et al. (38) developed dance programs with three weekly sessions over the same 12-week period, albeit with different approaches. The former included a modified Chinese square dance program designed to prevent falls, along with a combination of yoga and Korean dance, while the latter focused on the participation of elderly women.

Some interventions took a more specialized approach, such as Philip et al. (51), which incorporated 75-min community dance sessions for individuals with chronic respiratory diseases. Likewise, Holmerová et al. (52) implemented the EXDASE chair-based dance program, consisting of 75-min sessions once per week for three months. In a similar vein, Silva et al. (37) analyzed participation in weekly dance circles over the same period.

Regarding longer-duration programs, De Araujo & Da Rocha (36) examined participation in dance classes and musical gatherings over six months, a structure also followed by Lukach et al. (42). Meanwhile, Pandya (45) evaluated the combined intervention of Dance Movement Therapy (DMT) and Yoga (DMT + Y), whereas Zhao et al. (39) explored the impact of public dance sessions held three times per week for three months. In the case of Thumuluri et al. (44), the study analyzed improvisational dance classes using the IMPROVment® method, structured into twice-weekly sessions over eight weeks.

Some studies applied more traditional approaches, such as Douka et al. (19), which assessed a Greek dance-based program with two weekly sessions over 32 weeks, and Aguiñaga & Márquez (43), who examined a Latin dance program called BAILAMOS, structured with two sessions per week for 16 weeks.

Other studies focused on long-term interventions, allowing for an assessment of prolonged effects on participants' health. Lima & Vieira (35) examined a ballroom dance program that lasted one year, with two classes per week. Other studies explored interventions combining multiple disciplines, such as Poulos et al. (33), which evaluated courses integrating visual arts, photography, dance, theater, singing, and music, taught by professional artists over a period of 8–10 weeks, with weekly classes.

Overall, the reviewed studies highlight the diversity of approaches in implementing dance as a well-being strategy for the elderly. While short-term interventions primarily focus on immediate improvements in physical and emotional health, medium- and long-term programs tend to generate more sustained benefits, particularly in fall prevention, mental health enhancement, and the promotion of psychosocial development.

When we compared the types and durations of interventions with the observed outcomes, we found that short programs improved emotional and social domains, while long-term programs were associated with cognitive, physical, and mental health benefits.

### Types of research

Research on the relationship between dance and well-being in the elderly is reflected in the large number of studies and the diversity of approaches used. The most prominent group of studies explicitly aims to evaluate the impact of dance programs and activities (22, 33–35, 40, 42, 44, 45, 47–49, 52).exploratory studies (38, 39, 51) aimed at identifying patterns and generating hypotheses. Likewise, other studies focus on establishing or examining cause-and-effect relationships (19, 41, 46, 50). Additionally, some research falls into the comprehensive category, aiming for a holistic understanding of the subject (36, 37).

From a methodological perspective, a predominantly quantitative trend is evident, with 15 studies employing measurement batteries for data collection and analysis. This highlights a strong emphasis on the objective measurement of dance's effects on this population.

### Objectives and research instruments

The methodological strategies and instruments employed in these studies provide insight into how well-being was conceptualized and measured. This section outlines the tools used to assess physical, emotional, cognitive, and social outcomes, offering a clearer understanding of how dance-based interventions impact the multidimensional well-being of older adults.

All included studies used validated instruments to assess at least one dimension of well-being. Evaluative studies have developed rigorous methodologies to measure the impact of dance on the well-being of the elderly. For instance, Clifford et al. (47) assessed the feasibility of a dance program using instruments such as the Incidental and Planned Exercise Questionnaire (IPEQ), the Timed Up and Go (TUG) test, the 30-Second Sit-to-Stand (30CST) test, the Short Physical Performance Battery (SPPB), and the Single-Leg Stance (SLS) test to evaluate static postural control.

Similarly, Harrison et al. (22) examined the effectiveness of virtual ballet classes compared to Well-being sessions, employing self-report questionnaires and functional tests such as the Two-Minute Walk Test, the Timed Up and Go test, and the Mini-BESTest to assess balance and mobility confidence.

Also, some studies have adopted specialized methodologies to assess the impact of dance on elderly individuals with specific conditions. For instance, Pandya (45) investigated the effects of Dance Movement Therapy (DMT) and yoga on elders with Parkinson's disease, utilizing a range of assessment tools, including the Balance Confidence Scale, the Geriatric Anxiety Scale (GAS-10), and the Warwick-Edinburgh Mental Well-being Scale (WEMWBS). Additionally, the Mini-Mental State Examination (MMSE) was employed to evaluate cognitive function.

In a similar vein, Chipperfield & Stephenson (50) explored the influence of ballroom dancing on well-being and fall risk. Their research incorporated the CORE-GP questionnaire, the Falls Efficacy Scale–International (FES-I), and the Timed Up and Go Test (TUGT) to obtain a comprehensive understanding of its effects. Beyond these, other investigations have focused on refining assessment protocols, aiming to capture the multifaceted benefits of dance therapy in elderly populations.

Consequently, some other studies have explored the feasibility and impact of dance interventions in different contexts. For instance, Zhao et al. (39) examined the viability of public dance sessions for individuals with mild cognitive impairment and depressive symptoms, utilizing the Subjective Exercise Experience Scale (SEES) alongside heart rate monitoring to assess responses. Meanwhile, Philip et al. (51) focused on perceived well-being experiences among elders with chronic respiratory diseases, conducting semi-structured interviews and thematic analysis to gain deeper insights.

Based on this concern, Wang et al. (38) investigated the effects of Chinese square dancing in individuals with mild cognitive impairment, employing a combination of standardized assessments, including the Mini-Mental State Examination (MMSE) and the Montreal Cognitive Assessment (MoCA) for cognitive function, the Geriatric Depression Scale (GDS-15) for depressive symptoms, and the Berg Balance Scale (BBS) for postural stability.

Then, from an explanatory perspective, researchers have investigated how dance participation influences various aspects of life among elders. Han & Sa (41) analyzed the involvement of elderly women in dance activities and its impact on quality of life, health, and happiness, using participant interviews and on-site observations to gather qualitative insights. In another study, O'Toole et al. (46) explored the effects of dance on activity engagement, fall prevention, and overall well-being, employing the Falls Efficacy Scale and the EQ-5D-3l to assess perceived health and quality of life.

Finally, in the qualitative domain, several studies have explored the role of dance as a tool for health promotion and cultural expression. Silva et al. (37) examined circle dance as a strategy for health promotion, employing the qualitative research model by Schatzman and Strauss (Consolidated Criteria for Reporting Qualitative Research - COREQ) to structure their analysis. De Araujo & Da Rocha (36) investigated the meanings elders attribute to group leisure activities, specifically dance and musical gatherings. Their study applied participant observation, in-depth interviews, and ethnographic analysis to capture the participants' lived experiences.

Therefore, Lukach et al. (42) detailed the protocol of a multicenter study assessing the effects of African dance on cognition, mood, quality of life, and physical fitness among African American elders.

Literature reveals a limitation in the conceptualization of well-being, as many studies address it through specific dimensions, such as physical or emotional health, without integrating them into a holistic framework. To effectively assess well-being, a more comprehensive evaluation of interventions requires the adoption of multidimensional approaches.

One limitation related to the databases used was the exclusion of Google Scholar, due to its broad interdisciplinary coverage. This may have limited the inclusion of grey literatura (53). Finally, we highlight that a key limitation of this review is the restriction to studies that used the term well-being. We may have excluded studies by not using emotional, physical, social, or cognitive descriptors, which could limit the scope of the review. Some studies do not comprehensively assess well-being, and there is methodological heterogeneity. This underscores the need to implement tools that measure well-being from a multidimensional perspective.

## Conclusions

The reviewed studies confirm that dance practice has a positive impact on multidimensional well-being of older adults. From a physical perspective, significant improvements in strength, balance, and flexibility have been observed, contributing to fall prevention and the maintenance of functional autonomy. Emotionally and socially, dance has been shown to reduce anxiety and depression, enhance mood, and foster interpersonal interaction, promoting a sense of community and reducing loneliness. Cognitively, evidence suggests that dance stimulates memory and reduces the risk of cognitive decline, making it a key strategy for healthy aging.

Beyond these individual benefits, dance serves as an accessible and culturally meaningful tool for health promotion. Its integration into programs for elders allows for active participation in recreational activities that strengthen overall well-being. Due to its inclusive nature, dance can be practiced in open spaces, community centers, and clinical settings, extending its reach and facilitating its incorporation into daily life.

From a broader economic and political perspective, dance emerges as a cost-effective intervention that could be integrated into healthcare systems to improve the quality of life of aging populations. Furthermore, its implementation in both face-to-face and virtual settings through digital technologies has demonstrated therapeutic effects, creating new opportunities for accessibility and program expansion. However, challenges remain in promoting these initiatives, as their availability and implementation depend on individual practitioners and local policies.

For future research, randomized controlled trials are recommended to assess the effects of dance more accurately in larger samples. In addition, more analysis is needed on the economic and sociodemographic impact of dance in aging communities, comparing investment in dance programs as a preventive strategy vs. the costs associated with medical treatments for age-related diseases. Finally, motivational strategies should be explored to encourage participation among people who do not engage in regular physical exercise but who may find dance an attractive alternative for staying active. In conclusion, dance is a holistic practice that goes beyond physical activity, becoming a powerful tool for wellness.

## Data Availability

The original contributions presented in the study are included in the article/Supplementary Material, further inquiries can be directed to the corresponding author.
